# Behavior Changes of Patients With Type 2 Diabetes During the National Lockdown Due to the COVID-19 Pandemic

**DOI:** 10.7759/cureus.83741

**Published:** 2025-05-08

**Authors:** Rumyai Promsen, Niracha Chanwimol, Lukana Preechasuk, Pinyo Rattanaumpawan, Nuntakorn Thongtang

**Affiliations:** 1 Nursing, Siriraj Hospital, Bangkok, THA; 2 Medicine, Siriraj Hospital, Bangkok, THA

**Keywords:** covid-19 national lockdown, diets and physical activity, lockdown, medication adherence, type 2 diabetes

## Abstract

Background: We previously reported that the national lockdown led to worsening glucose control and increased systolic blood pressure in patients with type 2 diabetes (T2D). This study aimed to investigate the impact of the national lockdown on changes in diet and physical activity among these patients.

Methods: A questionnaire-based survey (N=100) was conducted to assess changes in dietary habits, physical activity, and medication adherence during the lockdown in a subset of patients from the Siriraj Diabetes Registry who attended a follow-up visit after the lockdown.

Results: The mean age of the participants was 60.3 ± 11.9 years, with 60% being female. Of 100 patients, changes in dietary patterns and physical activity were reported in 38 (38%) and 25 (25%) of patients, respectively. Self-reported weight gain occurred in 25 (25%) of participants. Medication adherence for anti-diabetic drugs, anti-hypertensive drugs, and lipid-lowering agents remained high, with 80 (80%) of patients reporting good compliance (defined as taking >80% of prescribed medications). No significant differences in medication adherence were observed during or after the lockdown compared to pre-lockdown levels.

Conclusions: The COVID-19 national lockdown in Thailand led to notable changes in dietary habits and physical activity among patients with T2D. However, medication adherence remained stable throughout the lockdown period.

## Introduction

Many countries have implemented national lockdown policies during the COVID-19 pandemic in the year 2020. During the lockdown in Thailand, travelling outside of one’s own province was restricted; thus, some patients have struggled to go to the hospital across provinces for a follow-up visit. Moreover, dining out at the restaurant and exercising in the park or at the gym were prohibited. The national lockdown in Thailand occurred only once in the year 2020, from 26 March 2020 to 17 May 2020. The duration of the lockdown was 53 days. We have previously reported that the Thailand national lockdown adversely affected glycemic control and clinic visitation in patients with type 2 diabetes (T2D) who used to come to follow-up regularly prior to the lockdown [1]. Moreover, there was a significant increase in mean systolic blood pressure after the lockdown [1]. The cause of worsening glucose and blood pressure control could be explained by changes in diets and lifestyles during the lockdown. A previous study that used data collected by questionnaire reported changes in diet and exercise during the lockdown in both type 1 and type 2 diabetes patients [2]. A questionnaire-based study collected in India during a national lockdown period reported that 13.7% of the patients with T2D changed their dietary intakes and that 14.5% changed their physical activities and watched more television at home [3]. Another study found that 23% of T2D patients reported eating more snacks [4]. The impact of these changes in diet and physical activity on metabolic parameters has been studied in type 1 diabetes and those using continuous glucose monitoring devices [5-8]; however, it has not been comprehensively investigated in T2D.

The aim of this study was to investigate changes in diet, physical activity, and diabetes self-management behaviors during the national lockdown among patients with T2D. The results of this study will help us better understand the healthcare dynamics of the patients and the healthcare setting and to develop a healthcare strategy for T2D to cope with the future pandemic.

## Materials and methods

Study protocol

This observational study was conducted at Siriraj Hospital, a tertiary care center located in Bangkok, Thailand. Adults with T2D from our center’s diabetes registry were screened for eligibility. The inclusion criteria were provided in the previous publication [1]: Briefly adult with T2DM who regularly attended scheduled follow-up appointments every two to three months during the year prior to the lockdown, had fasting plasma glucose (FPG) and HbA1c levels, and other metabolic parameters data from each follow-up prior to the start of the lockdown were recruited.

Of 825 patients who met the inclusion criteria, 600 patients were enrolled in the main study evaluating biochemical parameters before and after the lockdown [1]. A subset of patients in the diabetes registry was queried about their diets and physical activity during the national lockdown. Questionnaires were randomly given to 100 patients who satisfied the inclusion criteria and attended a follow-up during December 2020 to January 2021 using a convenient sampling method. The questionnaire inclusion criteria were patients with T2D, age ≥18 years, who attended a routine follow-up for their T2D, and were able to read and answer the questionnaire on their own. The exclusion criteria included individuals who were unable to respond to the questionnaire independently or were unwilling to participate. The questionnaire was written in Thai, the local language. The questionnaire was designed based on factors previously reported in the literature, as well as factors of interest related to diabetes self-management during the lockdown. It was reviewed by endocrinologists and pilot-tested on 10 participants to ensure clarity and appropriateness prior to the commencement of the study. The final version was self-administered during routine follow-up visits. It consisted of 30 multiple-choice questions in Thai. It consisted of two domains: A) sociodemographic details (e.g., age, gender, city, and educational qualification and B) impact of national lockdown on diabetes care (e.g., access to health care services, glucometer at home, changes in the eating habits and physical activity, changes in drinking and smoking habits, and changes in medication adherence). The questions were designed to compare the situation “during” the lockdown to “before and after” the lockdown. The English-translated version of the questionnaire is provided in the appendices. The study protocol was approved by the Siriraj Institutional Review Board (COA no. Si 873/2563), and all subjects provided written informed consent. All methods were performed in accordance with the Declaration of Helsinki.

Sample size calculation

A previous study reported that 44% of patients changed their dietary pattern as a result of the lockdown [4]. Using these data and an allowable error of 0.1, a sample size of 95 T2D was required. This figure was then rounded up to 100 subjects.

Statistical analysis

Descriptive statistics were used to summarized patient characteristics. Categorical variables were expressed as a number and/or percentages, while continuous variables were expressed as mean ± standard deviation (SD). Paired t-test was used to compare parameters before and after the lockdown. Independent samples t-test was used to compare parameters between groups. All statistical analyses were performed using Statistical Product and Service Solutions (SPSS, version 18.0; IBM SPSS Statistics for Windows, Armonk, NY).

## Results

Baseline characteristics of the study patients

Six hundred T2D patients who attended regularly scheduled follow-up appointments every two to three months at our center during the year prior to the Thailand COVID-19 national lockdown were enrolled in the main study. The mean age of patients was 63.7 ± 11.3 years, and 64.8% were female. Close to half (49.7%) of study patients were aged ≥65 years old. Good glycemic control, which was defined as HbA1C <7% (<53 mmol/mol), was found in 25.7% of the patients. Among the 100 patients invited to complete the questionnaires, 60 subjects (60%) were female. The mean age was 60.3 ± 11.9 years. Forty percent of study patients were aged≥65 years old. The baseline characteristics of the patients who answered the questionnaire were not significantly different from the patients in the main study.

Of 100 patients, 55 subjects (55%) of the respondents lived in Bangkok, where Siriraj Hospital was located, and the others lived in other provinces of Thailand. Sixty-nine (69%) respondents reported having an education level of less than 12 years, and 31% were university graduates. Twenty-eight percent were retired, 18% were unemployed, and 54% were employed. Thirty-two percent reported feeling that it was difficult to travel to the hospital during the lockdown, and 44% reported a fear of contracting COVID-19 at the hospital during a follow-up visit for T2D care (Table 1).

**Table 1:** Baseline characteristic of the patients who completed the questionnaire (N=100) Data presented as mean ± standard deviation or %; *Fasting glucose level and HbA1c data are available in 70 subjects.

Parameters	Results	
		Age	60.3 ± 11.9 years	
Current Residence		
In the same province as the hospital	55 (55%)	
Different provinces as the hospital	45 (45%)	
Education Level		
Primary education	24 (24%)	
Secondary	45 (45%)	
University graduate	31 (31%)	
Occupation		
Retired	28 (28%)	
Unemployed	18 (18%)	
Employed	54 (54%)	
Fasting glucose level (mg/dL)*	135.7 ± 36.1	
HbA1c (%)*	7.2 ± 1.5	

Questionnaires evaluating dietary patterns and physical activity during the lockdown

**Figure 1:**
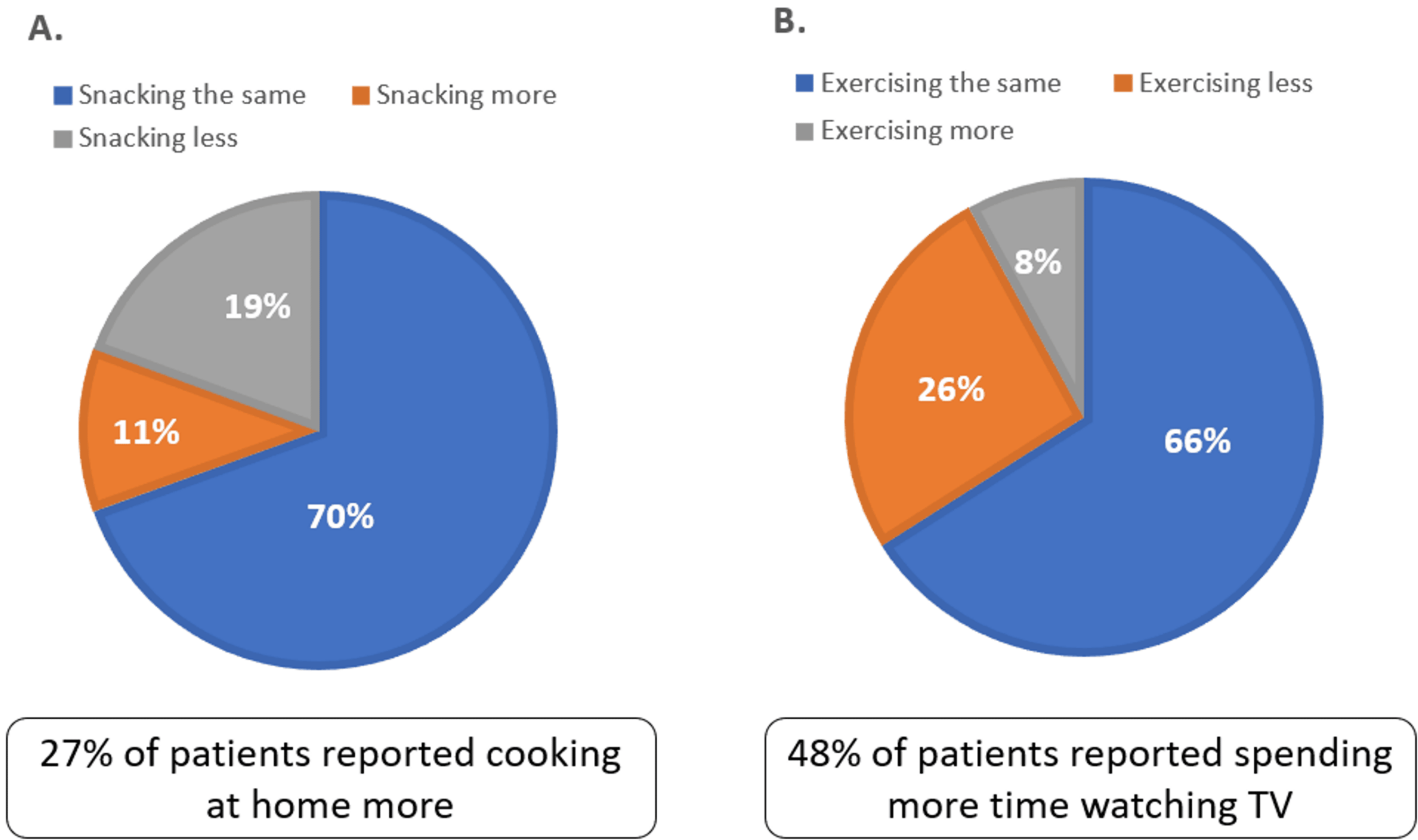
Self-reported snacking frequency and frequency of exercise A. Frequency of snacking B. Frequency of exercise

Thirty-eight subjects (38%) of the subjects reported a change in dietary habits by either changing snacking frequency or changing the amount of food intake. Among these patients (N=38), four patients (10.5%) reported snacking more often, five patients (13.2%) increased the amount of food intake, and seven patients (18.4%) reported less snacking or decreased the amount of food intake (Figure 1A). However, 27% of the patients reported cooking at home more.

Among the 100 subjects queried, 59 subjects (59%) had a baseline of sedentary lifestyle before the lockdown defined as exercise <50 minutes/week. However, 26 subjects (26%) reported that they exercise even less during the lockdown (Figure 1B). During the lockdown, 48 subjects (48%) of the patients reported spending more time watching television or receiving news from media, and 51 subjects (51%) reported feeling more stressed. Body weight increase was self-reported in 25% of the patients. Moreover, worsened glucose control was self-reported in 31% of patients during the lockdown, and in 21% of patients after the lockdown. Only 34% of patients reported having glucometers at home.

Drug adherence during the lockdown

Drug compliance assessed were anti-diabetic drugs, anti-hypertensive drugs, and lipid-lowering agents. Each drug classes were asked separately. The compliances of all drug classes were non-significant different between before and during the lockdown. Eighty-percent of the patients were in good compliance with self-reported drug compliance >80% of their prescribed medications. There was no significant change in drug compliance during and after a lockdown as compared to pre-lockdown.

## Discussion

Type 2 diabetes was reported to be a risk factor for severe disease in patients with SARS-CoV-2 virus infection [9,10]. Well-controlled blood glucose correlated with improved outcomes in T2D infected with the virus [11]. Social distancing and national lockdowns are strategies that are employed to help control the spread of viruses. Due to limits on outside-of-the-house activities during a lockdown, many studies have reported changes in dietary patterns such as increased snacking frequency, changes in the amount or type of food eaten, reduced physical activity, and increased body weight [12-15]. These factors alone or together could exert negative health impacts, especially in patients with metabolic syndrome and T2D. Similar to the previous study, we found that 38% and 25% of the patients, respectively, reported changes in snacking frequency and physical activity during the lockdown. In addition, weight gain was self-reported in 25% of the study patients from the questionnaire. These could explain a significant worsening in glycemic control after the national lockdown in our main study, which was reported earlier [1]. However, most studies in type 1 diabetes who are well educated in diabetes self-management and were using continuous glucose monitoring devices during the lockdown have reported improvement in glycemic control [5,6,16] or a reduction in hypoglycemia events [8]. Although a continuous glucose monitoring device is not currently widely used among T2D, the findings strengthen the importance of self-management education and self-monitoring of glucose. A study in T2D who had access to a glucometer and teleconsultation had better glucose control during a lockdown [17]. However, our questionnaire data revealed that only 34% of patients own a glucometer to perform self-monitoring of glucose. Thus, self-monitoring of blood glucose should be emphasized for the better care of patients with diabetes.

Psychological stress during a lockdown has been reported in many studies, including in patients with T2D [2,3,18]. Fifty-one percent of the patients reported having more stress during the lockdown. Increased psychological stress during social isolation or stress due to the fears about the pandemic itself may explain our finding of a significant increase in systolic blood pressure in our main study [1]. This is because anti-hypertensive medication adherence did not differ significantly during or after the lockdown compared to baseline from this questionnaire study.

Although there was a significant worsening in glucose and blood pressure control during the lockdown, plasma LDL-cholesterol was not adversely affected. A systematic review that investigated dietary habits during a lockdown reported increased snacking frequency, eating more home-prepared food, and eating less fast food [13]. We also found that 37 (37%) study patients reported cooking at home more. The result of eating more home-prepared foods and less fast-food consumption may also explain the reduction in plasma LDL-cholesterol levels. T2D patients in this study were on statins, and the questionnaire data showed medication compliance to be unchanged or slightly better during the lockdown. This may explain the observed slight improvement in lipid controls found in our previous study [1].

Medication adherence did not differ can be because the patients were the population who had regular clinic visitation to the hospital prior to the lockdown. Self-care and disease awareness in these patients are better.

Our study has some limitations. First, we did not survey the data during the national lockdown; the data were based on self-reported changes in behaviors after the lockdown was lifted. However, the patients were asked to compare their behaviors before and after the lockdown. Second, the use of convenience sampling may have introduced selection bias. Nonetheless, this method was chosen due to practical constraints encountered during the pandemic. Future studies with larger sample sizes should be conducted to validate and generalize our findings.

## Conclusions

In summary, this study found that the national lockdown in Thailand during the COVID-19 pandemic led to notable lifestyle changes in patients with T2D. There were significant alterations in dietary patterns and a marked increase in physical inactivity. As a result, 25 (25%) participants experienced weight gain during the lockdown period. Despite these changes, medication adherence remained stable, indicating that patients continued to follow their prescribed treatment regimens. These findings highlight the need for supportive measures to help patients with chronic conditions maintain healthy behaviors during public health emergencies.

## Appendices

Questionnaire

“The Impact of the COVID-19 Lockdown on Glycemic Control and Metabolic Outcomes in Patients with Type 2 Diabetes Mellitus”

Objective of the Research Project

To study the impact of city lockdown measures, implemented to contain the spread of COVID-19, on dietary behaviors, physical activity, exercise, and medication adherence among patients with type 2 diabetes. The questionnaire takes approximately 5-7 minutes to complete. Please answer all questions. Circle or mark an X on the number that corresponds to your answer.

We sincerely thank all participants. The responses collected are for research purposes only and will not affect your current medical treatment in any way.

Respondent Information

1. Age: …………….. years

2. Gender:                  1) Male                                       2) Female

3. Place of origin    1) Bangkok                            2) Outside Bangkok

4. Current residence: 1) Bangkok                              2) Outside Bangkok

5. Medical coverage:

 1) Universal coverage / Social Security at Siriraj Hospital

 2) Universal coverage / Social Security at other hospitals

 3) Government official / State enterprise

 4) Self-pay

 5) Private insurance / Company reimbursement

6. Education level:

 1) No formal education  2) Primary education      3) Lower secondary

 4) Upper secondary            5) Vocational / Technical   6) Bachelor’s degree or higher

7. Current occupation:

 1) Retired                        2) Unemployed                3) Own business / Vendor

 4) Government official / State enterprise employee         5) Farmer

 6) Company employee     7) Healthcare personnel     8) Other (please specify):……………………

8. Do you believe that diabetic patients will have more severe symptoms from COVID-19 compared to non-diabetics?

 1) Yes 2) No 3) Not sure

9. Have any of your close contacts been at risk of COVID-19 infection?

 1) Yes 2) No 3) Not sure

Impact of Lockdown (March 26 - May 17, 2020) on Diabetes Care

10. Was it more difficult for you to access hospital services during the lockdown?

 1) Yes 2) No

11. Were you afraid to visit the hospital due to concerns about contracting COVID-19?

 1) Yes 2) No

12. Did your dietary habits change during the lockdown?

 1) Yes 2) No (If no, skip to Question 16)

13. Daily food intake during lockdown:

 1) Increased 2) Remained the same 3) Decreased

14. Number of meals/snacks per day during lockdown:

 1) Increased 2) Remained the same 3) Decreased

15. Home cooking during lockdown:

 1) Increased 2) Remained the same 3) Decreased

16. Weight after lockdown (post-June) compared to pre-lockdown:

 1) Increased 2) Remained the same 3) Decreased

17. Pre-lockdown exercise frequency:

 1) <50 minutes/week 2) 50-149 minutes/week 3) ≥150 minutes/week

18. Physical activity/exercise during lockdown (March-May 2020):

 1) Increased 2) Remained the same 3) Decreased

19. Time spent watching TV, listening to news, or reading news during lockdown:

 1) Increased 2) Remained the same 3) Decreased

20. Stress levels during the COVID-19 outbreak:

 1) Increased 2) Remained the same 3) Decreased

21. Sleep quality during the COVID-19 outbreak:

 1) Same 2) Worsened

22. Do you smoke?

 1) No 2) Yes

 If yes, smoking frequency during lockdown:  1) Increased 2) Remained the same 3) Decreased

Glycemic Control and Medication Adherence

23 Did your blood sugar levels increase during the lockdown?

 1) Yes 2) No 3) Not sure

24. Did your blood sugar levels increase after the lockdown was lifted?

 1) Yes 2) No 3) Not sure

25. Do you have a home glucose meter?

 1) Yes 2) No (If no, skip to Question 28)

26. Frequency of blood glucose testing before lockdown:

 1) >4 times/month 2) 1-2 times/month 3) <1 time/month

27. Frequency of blood glucose testing during lockdown:

 1) Increased 2) Remained the same 3) Decreased

28. Did you experience hypoglycemia (low blood sugar) during lockdown?

 1) No 2) Yes

 If yes, frequency of hypoglycemia during lockdown:

 1) Increased 2) Remained the same 3) Decreased

Medication Adherence

**Table 2:** 29. Medication adherence during lockdown (March–May 2020) Mark an X in the relevant boxes for each type of medication. How much of your prescribed medication did you take?

% taken	Type of medications		
	BS lowering	BP lowering	Lipid lowering agents
1) 100%			
2) >80%			
3) 50-80%			
4) <50%			
5) Not currently prescribed any medication by doctor			

**Table 3:** 30. Current medication adherence (Please mark X in the relevant boxes): How much of your prescribed medication are you currently taking?

% taken	Type of medications		
	BS lowering	BP lowering	Lipid-lowering agents
1) 100%			
2) >80%			
3) 50-80%			
4) <50%			
5) Not currently prescribed any medication by doctor			
